# Thermostable adenosine 5′-monophosphate phosphorylase from *Thermococcus kodakarensis* forms catalytically active inclusion bodies

**DOI:** 10.1038/s41598-021-96073-5

**Published:** 2021-08-19

**Authors:** Sarah Kamel, Miriam C. Walczak, Felix Kaspar, Sarah Westarp, Peter Neubauer, Anke Kurreck

**Affiliations:** 1 Chair of Bioprocess Engineering, Technische Universität Berlin, Straße des 17. Juni 135, 10623 Berlin, Germany; 2 BioNukleo GmbH, Ackerstraße 76, 13355 Berlin, Germany

**Keywords:** Biochemistry, Biotechnology, Microbiology

## Abstract

Catalytically active inclusion bodies (CatIBs) produced in *Escherichia coli* are an interesting but currently underexplored strategy for enzyme immobilization. They can be purified easily and used directly as stable and reusable heterogenous catalysts. However, very few examples of CatIBs that are naturally formed during heterologous expression have been reported so far. Previous studies have revealed that the adenosine 5′-monophosphate phosphorylase of *Thermococcus kodakarensis* (*Tk*AMPpase) forms large soluble multimers with high thermal stability. Herein, we show that heat treatment of soluble protein from crude extract induces aggregation of active protein which phosphorolyse all natural 5′-mononucleotides. Additionally, inclusion bodies formed during the expression in *E. coli* were found to be similarly active with 2–6 folds higher specific activity compared to these heat-induced aggregates. Interestingly, differences in the substrate preference were observed. These results show that the recombinant thermostable *Tk*AMPpase is one of rare examples of naturally formed CatIBs.

## Introduction

Heterologous expression of genes in *Escherichia coli* often leads to intracellular aggregation of the target overproduced protein which are called inclusion bodies (IBs). The seed of IB formation is the presence of unproperly folded protein which is induced by several factors such as uncontrolled/unfavored growth pH and/or temperature, oxidative stresses, high rate of heterologous proteins expression (using strong expression vectors or high inducer concentrations) which exceeds the protein folding. Additionally, the failure of the cell to post-translationally modify the protein or to form the inter- and intra-subunit disulphide bonds are reasons for IB formation^1–6^.

IBs are currently defined as an amorphous mixture of amyloid-like cross molecular beta sheets and native (like) folded protein structures that are substantially active and could be further used as such without the need to re-fold^7–9^. They, additionally, often contain the aggregated protein in high concentration with relatively little contamination by other intracellular proteins. For this reason, intentional aggregation of the target protein and purification of the resulting IB is a widely used strategy for product concentration and crude purification in the pharmaceutical industry. Nonetheless, laborious downstream purification, solubilization and refolding steps are typically required to obtain the target protein in the correct (active) folding state^2,5,10^.

Catalytically active inclusion bodies (CatIBs) offer a valuable alternative as they can be produced in *E. coli* without the need for laborious solubilization. As such, CatIBs can be used as immobilized enzymes, thus increasing the interest in this class of protein aggregates in recent years^1,2,11^. The application of CatIBs offers several advantages compared to soluble or synthetically immobilized enzymes. These include compatibility with aqueous and non-aqueous media, straightforward and cheap purification, reusability and no loss of activity due to immobilization^1^.

CatIBs are usually engineered by the addition of small peptide tags or aggregation-inducing protein domains to a protein of interest. These folding active centers guide the protein to accumulate in inclusion bodies. While most publications so far describe such engineered protein variants forming CatIBs^12–16^, only few examples have been reported of naturally occurring proteins developing CatIBs during overexpression in *E. coli*^17–20^.

**Figure 1:**
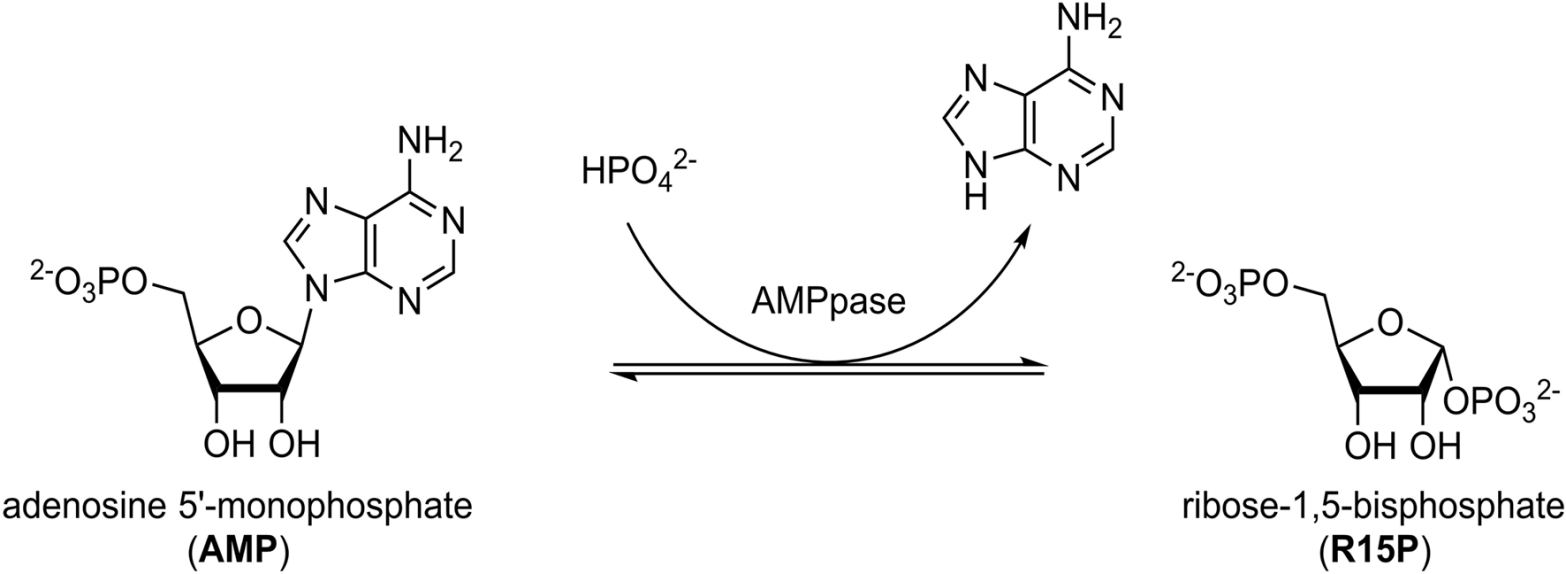
The reaction catalyzed by adenosine 5′-monophosphate phosphorylases (AMPpases). The *N*-glycosidic bond of adenosine 5′-monophosphate (AMP) is cleaved in the presence of inorganic phosphate yielding adenine and ribose-1,5-bisphosphate (R15P).

Herein, we describe the isolation of in-vivo formed CatIBs and in-vitro formed heat-induced aggregates (HIA) of *Thermococcus kodakarensis* adenosine 5′-monophosphate phosphorylase (*Tk*AMPpase). This enzyme was first described in 2007 as a biocatalyst involved in a previously undescribed metabolic pathway in archaea^21–23^. This pathway is involved in supplying the ribose moiety of 5′-mononucleotides (NMP) to the central carbon metabolic pathway(s)^21^. *Tk*AMPpase phosphorolytically cleaves the *N*-glycosidic bond of NMP in the presence of inorganic phosphate yielding the corresponding nucleobase and ribose-1,5-bisphosphate (R15P; Fig. 1)^21–23^. The enzyme has previously been misannotated as a thymidine phosphorylase until Sato et al.^21^ recognized its function in the AMP metabolic pathway in archaea. Although *Tk*AMPpase’s primary structure is closely related to thymidine phosphorylases, it’s quaternary structure was reported to form unusual multimers (> 40-mers)^23^. Previous functional studies showed that the enzyme is highly active at 85 °C and could withstand high temperatures (60–85 °C) for at least 24 h^22–24^. Aono et al.^22^ additionally showed that the enzyme accepts all natural NMPs and their corresponding 2′-deoxy-NMP (dNMP) with preference for cytidine 5′-monophosphate (CMP).

While we were studying this enzyme in more detail, we discovered that it is prone to aggregation both during expression and after exposure to heat in cell crude extract which led us to develop a purification method to obtain the insoluble protein as CatIBs. Interestingly, insoluble preparations of *Tk*AMPpase retained high activity with all natural 5′-mononucleotides, opening new avenues for the application of this enzyme as a heterogenous biocatalyst.

## Materials and methods

### General information

All chemicals were of analytical grade or higher and purchased, if not stated otherwise, from Sigma-Aldrich (Steinheim, Germany), Carl Roth (Karlsruhe, Germany), TCI Deutschland (Eschborn, Germany), NMPs were purchased from Carbosynth (Berkshire, UK) or VWR (Darmstadt, Germany). All experiments were repeated for at least 3 times unless specified otherwise.

### Cloning and expression of *Tk*AMPpase

The *Thermococcus kodakarensis* gene sequence obtained from the national center for biotechnology information (NCBI) database coding AMPpase protein with accession number WP_011249307.1 was codon-optimized for expression in *E. coli* and obtained by gene synthesis (GeneArt, Regensburg, Germany). The *Tk*AMPpase gene was then cloned via NdeI/HindIII digestion (FastDigest restriction endonucleases, Fermentas, Vilnius, Lithuania) and ligation (T4 DNA Ligase, Roche) into an expression vector (pKS-2) modified from pCTUT7 as described previously^25,26^.

The *N*-terminally His_6_-tagged *Tk*AMPpase was expressed in *E. coli* BL21 in shake flask using 50 mL of EnPresso B medium (Enpresso, Berlin, Germany) at 30 °C. As recommended by the manufacturer, after overnight incubation of the main culture, expression was induced using IPTG with a final concentration of 1 mM. Each 50 mL culture yielded 1.8–2.2 g cell pellet. The harvest OD_600_ was on an average equal to 43.

An OD_600_ = 5 cell pellet sample (25 mg wet weight) was used to test the protein expression using BugBuster Protein Extraction Reagent (Merck, Darmstadt, Germany). For cell lysis, the cell pellet was incubated for 30 min with 300 μL of BugBuster reagent complemented with lysozyme (final conc. of 50 µg mL^−1^), DNase (final conc. of 1 µg mL^−1^) and MgCl_2_ (final conc. of 1 mM). The cell lysate was centrifuged (4 °C, 16,000*g* for 15 min) to separate the soluble (S) and insoluble (IS) protein fractions. 5 µL of the protein fractions were analyzed using 12% SDS–polyacrylamide gels according to standard protocols^27^ and a protein marker ranging from 10 to 200 kDa (New England BioLabs, MA, USA). The percentage of soluble and insoluble fractions was quantified from the SDS-polyacrylamide gels by densitometric analysis (ImageJ software (National Institute of Health, USA, http://www.imagej.nih.gov/ij))^28^.

To purify *Tk*AMPpase three different methods were applied which are summarized in Supp. Table 1. To obtain heat induced aggregates and purified soluble protein the cells were disrupted by French Press and the soluble fraction was either heat-treated or transferred to Ni-NTA affinity chromatography. To avoid the disruption of the active IBs and an associated inactivation French Press cell disruption was not used for CatIB preparation and therefore cell disruption was performed by enzymatic lysis and sonification.

### Preparation of *Tk*AMPpase as soluble protein in crude extract

For cell disruption, each 1 g of cell pellet (wet weight) was resuspended in 5 mL 0.1 M Tris–HCl buffer containing 1 mM EDTA (pH 7). In each experiment about 4 g cell pellet (wet weight) was used. Cells were mechanically lysed by French Press to obtain soluble *Tk*AMPpase. The French press was used for five consecutive cycles at 900–1000 bar. After each cycle, a sample of 1 mL was taken for further analysis. Following cell disruption, the suspension was supplemented with a solution of 1.5 M NaCl, 60 mM EDTA, 6% Triton-X100 (pH 7) and 0.1 mM PMSF (half of the remaining volume each) and incubated for 30 min on ice. Insoluble protein was separated from the soluble one by centrifugation at 4 °C, 8000*g* for 10 min (Supp. Fig. 1). 5 µL of both soluble and insoluble protein fractions were analyzed by SDS-PAGE. The insoluble fraction was treated with standard SDS loading buffer containing 80 mM urea according to the standard protocols^27^. The soluble protein fraction obtained after the fifth French press cycle was stored at 4 °C for until further use. French Press cell disruption experiment were repeated four independent times.

### Purification of soluble *Tk*AMPpase using affinity chromatography

Each 1 g of cell pellet was resuspended in 5 mL lysis buffer (50 mM sodium phosphate, 300 mM NaCl, 10 mM imidazole, pH 7) complemented with lysozyme (final conc. of 50 µg mL^−1^), DNase (final conc. of 1 µg mL^−1^) and MgCl_2_ (final conc. of 1 mM). The cell suspension was incubated at room temperature (25 °C) for 30 min. Afterwards, cells were mechanically lysed by French Press at 900–1000 bar for five consecutive cycles. The lysed cells were centrifuged at 4 °C, 8000*g* for 10 min to separate the soluble and the insoluble fraction. The soluble fraction was loaded to a 5 mL Ni-NTA column (Jena biosciences, Jena, Germany). The flowthrough was collected and loaded to a second 5 mL Ni-NTA column to maximize the amount of soluble protein obtained. The columns were washed three times using washing buffer (50 mM sodium phosphate, 300 mM NaCl, 20 mM imidazole, pH 7). Finally, the protein was eluted using elution buffer (50 mM sodium phosphate, 300 mM NaCl, 250 mM imidazole, pH 7) in five fractions, 2.5 mL each. 5 μL of each fraction of the purification process was analyzed on 12% SDS-polyacrylamide gels. The insoluble fraction was treated with standard SDS loading buffer containing 80 mM urea according to the standard protocols^27^.

### Purification of CatIBs

For the isolation of CatIBs from *E. coli* cultures, 1 g of cell pellet was resuspended in 5 mL of 0.1 M Tris–HCl buffer (pH 7) containing 1 mM EDTA and 1.5 mg mL^−1^ lysozyme. The cell suspension was then incubated for 30 min at room temperature (approximately 25 °C) followed by mechanical lysis on ice using sonification for 5 min with 30% power input and 30 s on/off intervals. The mixture was then treated with DNase (final concentration of 50 µg) in the presence of 3 mM MgCl_2_ and 0.1 mM PMSF. After incubation at 37 °C for 30 min, a solution of 1.5 M NaCl, 60 mM EDTA and 6% Triton-X100 (pH 7) was added (half of the current volume). The cell suspension was then incubated for 30 min on ice. To evaluate the cell lysis efficiency, a 100 µL sample was taken, the soluble (S) and insoluble proteins (IS) were separated by centrifugation (4 °C, 16,000*g* for 15 min). For the SDS-PAGE analysis 2 µL of 1:10 dilution was loaded on the gel.

The IBs were collected by centrifugation of the cell suspension (4 °C, 8000*g* for 10 min), followed by three successive washing steps each with 40 mL of 0.1 M Tris–HCl containing 20 mM EDTA (pH 7). 500 µL sample was taken after each washing step and 10 µL from each (W1, W2 and W3) was loaded on the SDS polyacrylamide gel. The isolated IBs were stored at 4 °C until further use. For SDS-PAGE analysis, soluble protein fraction and wash samples were treated with SDS loading buffer, while insoluble protein fraction and IB were treated with SDS loading buffer containing 80 mM urea according to standard protocols^27^. For the purpose of detection, 2 µL of IB in a 1:16 dilution was loaded on the SDS-PAGE. For activity assays 0.2 g of isolated IBs were resuspended in 500 µL MOPS buffer (50 mM, pH 7.5).

### Aggregation of *Tk*AMPpase from the crude extract by heat

1.5 mL of the soluble *Tk*AMPpase obtained after the fifth French press cycle was incubated at different temperatures (room temperature (approximately 25 °C), 40, 50, 60, 70, 80 and 90 °C) to induce protein aggregation (Supp. Fig. 1). 200 µL samples were taken at 15, 30, 45 and 60 min and stored on ice. The heat-induced aggregates (HIAs) were collected by centrifugation (4 °C, 16,000*g* for 10 min). The obtained aggregates were resuspended in 200 µL solution of 1.5 M NaCl, 60 mM EDTA, 6% Triton-X100 (pH 7). For SDS-PAGE analysis, 2 µL of 1:10 dilutions of soluble and the HIAs were treated with standard SDS loading buffer and SDS loading buffer containing 80 mM urea, respectively, according to standard protocols^27^. Using densitometric analysis (ImageJ software (National Institute of Health, USA, http://www.imagej.nih.gov/ij))^28^ the relative amount of HIAs was calculated as a percentage of the total *TkAMPpase* protein (soluble and insoluble) per time point according to the formula below

$$Percentage\, of\, HIA=\frac{density\, of\, TkAMPpase\, HIA\, band}{density\, of\, TkAMPpase\, soluble\, band+density\, of\, TkAMPpase\, HIA\, band}\times 100$$

Standard deviations were calculated from three independent experiments. For analysis of the specific activity with 5′-mononucleotides, 0.2 g of HIAs were resuspended in 500 µL MOPS buffer (50 mM, pH 7.5).

### Analysis of the thermostability of His-tag purified *Tk*AMPpase

1.5 mL of 1 mg mL^−1^ of the his-tagged purified *Tk*AMPpase was incubated at 60 or 80 °C for 1 h or 24 h. Samples were cooled on ice for 30 min and centrifuged at maximum speed for 30 min. 500 µL samples were taken, 10 µL were loaded on 12% SDS-polyacrylamide gels and *TK*AMPpase was quantified with densitometric analysis of the polyacrylamide gels using ImageJ software (National Institute of Health, USA, http://www.imagej.nih.gov/ij)^28^. The percentage of the formed aggregation is calculated relative to the density of the sample not treated with heat (which is set to 100%).

### Quantification of the *Tk*AMPpase CatIB and HIA

BSA was used as a standard to determine *Tk*AMPpase concentrations from different preparations (HIAs and IBs) using SDS-PAGE. Three different concentrations of BSA (0.5, 0.25 and 0.1 mg mL^−1^) were loaded on 12% SDS–polyacrylamide gels in duplicates. The concentrations were chosen to be in the linear detection range. The densities of the bands were analyzed using ImageJ software (National Institute of Health, USA, http://www.imagej.nih.gov/ij)^28^ and standard curves were generated (Supp. Fig. 2). Serial dilution of the HIAs and CatIBs of *Tk*AMPpase were loaded on SDS–polyacrylamide gels and the band densities were quantified using the BSA standard curve as a reference (Supp. Fig. 3 and 4).

### Protein quantification by a modified Bradford assay

For the quantification of the total protein and the His-tag purified protein, Roti-Nanoquant reagent (Carl Roth, Karlsruhe, Germany) was used. BSA was used as a standard with 9 different concentrations ranging between (0–100 µg mL^−1^). All samples were properly diluted to fit within the range of the standards. The assay was performed in 96 deep-well plate and the absorbance was measured at 450 and 590 nm as recommended by the manufacturer. All the measurements were performed at least in triplicates.

### Enzyme activity assay

The specific activity of the His-tag purified, HIAs and CatIBs of *Tk*AMPpase were tested with natural NMPs. A proper dilution was used in which the enzyme OD_260_ did not exceed 0.1. Reactions were performed with 2 mM nucleotide and 50 mM phosphate in 50 mM MOPS buffer (pH 7) in the presence of 50–100 µg mL^−1^ enzyme at 80 °C. Regular samples were taken to monitor the initial rates of the reactions (< 12% product formation). The minimum reaction time was 2.5 min for His-tag purified *Tk*AMPpase and the maximum was 40 min for HIAs obtained at 90 °C. Samples were stopped and quenched by adding 40 µL of reaction mixture to 460 µL of 0.5 M NaOH (CMP-containing reactions) or 0.1 M NaOH (AMP-, GMP- and UMP-containing reactions). The nucleobase/nucleotide ratio in each sample was obtained via deconvolution of the experimental UV absorption spectra using suitable reference spectra obtained under the same conditions as described previously^29,30^. The specific activities were calculated in units (U) per mg enzyme, where one U is the conversion of 1 µmol substrate per minute under the conditions stated above.

## Results and discussion

### Heterologous expression of *Tk*AMPpase

**Figure 2:**
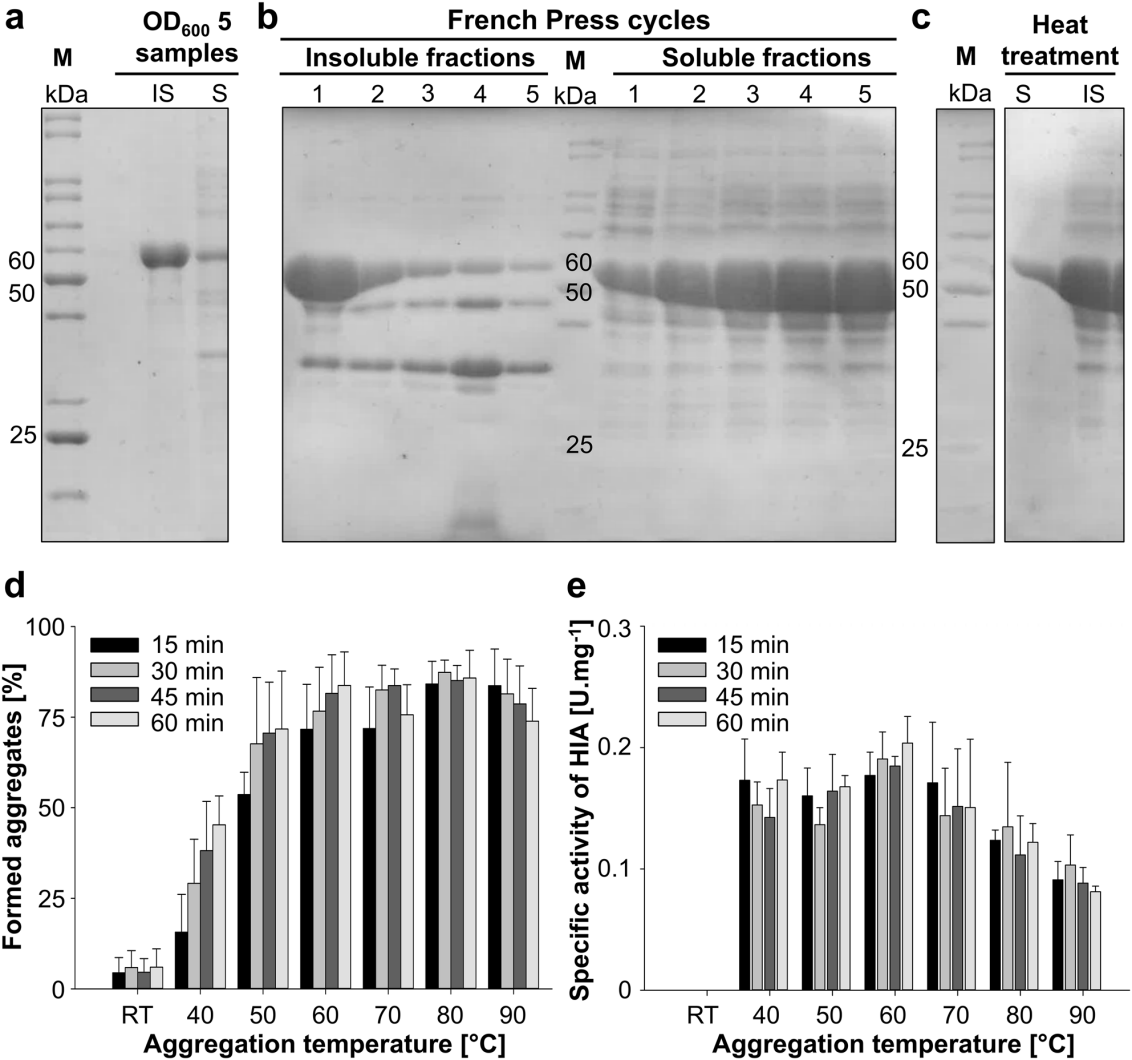
Solubilization and formation of heat-induced aggregates (HIAs) of *Tk*AMPpase. (**a**) SDS-PAGE analysis of 4 µL both soluble (S) and insoluble (IS) protein fractions of OD_600_ = 5 samples after the expression of *Tk*AMPpase in *E. coli* using Enpresso B medium. The *Tk*AMPpase (54 kDa) is mainly detected in the insoluble fraction (IS) after cell disruption using BugBuster Protein Extraction Reagent. M: protein marker. (**b**) 5 µL of the protein fractions obtained from French Press cell disruption at 900 bar analyzed by SDS-PAGE. (**c**) The fifth French Press fraction of the soluble protein was further purified by heat treatment at 80 °C for 30 min which resulted in a significant re-aggregation of the protein and its accumulation in the insoluble fraction. The marker (M) lane and the sample lanes are rearranged from the same gel. The complete SDS-PAGE from (**a**), (**b**) and (**c**) are shown in the supplementary material. (**d**) The induction of aggregation by heat at different temperatures within 1 h. Room temperature (approximately 25 °C) was used as a negative control to examine spontaneous aggregation. Chart is generated based on densitometric analysis of SDS–polyacrylamide gels using ImageJ software. (**e**) The specific activity of all aggregates was determined with 2 mM CMP and 50 mM phosphate in 50 mM MOPS buffer (pH 7.5) at 80 °C.

To study *Tk*AMPpase in more detail, we expressed *Tk*AMPpase using an IPTG-inducible expression vector and an EnPresso B medium which mimics the glucose limited fed-batch cultivation process in shake flasks. This ensures a controlled glucose release, bacterial growth and subsequently increases the efficiency of target gene expression. 50 mL culture yields around 1.9–2.2 g of cell pellet (wet weight). The recombinant *Tk*AMPpase (54 kDa) was expressed mainly in the insoluble fraction as detected in samples (OD_600_ = 5) taken after expression (Fig. 2a, Supp. Fig. 11). On average, about 80% of the totally expressed *Tk*AMPpase was found in the insoluble fraction as calculated using densitometric analysis. We attributed this to *Tk*AMPpase forming unusually large multimers (> 40-mers)^23^. This multimeric structure was described to be linked together by 10 amino acid residues in the C-terminal domain. Additionally, the protein N-terminus (84 amino acid) contributes to the multimer formation. Although the relation between the protein oligomerization state and its solubility is not well reported, there are few reports suggesting that multimeric proteins such as l-asparaginase and beta-galactosidase (both tetramer) tend to aggregate during heterologous expression^8,20^. In previous reports, *Tk*AMPpase was described to be expressed in the soluble protein fraction^22,23^, however, the insoluble fractions were not analyzed in these reports (personal communication). Accordingly, the multimeric structure of *Tk*AMPpase might explain its aggregation during expression. Nonetheless, it is also plausible that expression conditions such as expression vector, strain, medium and others may have an impact on the solubility of the expressed protein^5,31^. The authors of the previous work have used a pET21a vector, a BL21 (DE3) expression strain and LB medium at 37 °C^22,23^, which might have had a positive impact on the soluble expression of *Tk*AMPpase.

### Optimized preparation of soluble *Tk*AMPpase

Since *Tk*AMPpase was obtained primarily as insoluble protein, we developed a high-pressure cell disruption protocol using a French press. Pressure of up to 2 kbar has previously been reported to disrupt the oligomerization of some proteins such as glyceraldehyde-3-phosphate dehydrogenase, lactate dehydrogenase, malate dehydrogenase or tryptophan synthase, which led us to hypothesize that a similar strategy might be employed to solubilize *Tk*AMPpase^32,33^. Our results showed that after five consecutive cell disruption cycles at 900 bar, the amount of the recombinant *Tk*AMPpase in the soluble fraction increased to 30% after the fifth French Press cycle with a stepwise increase after each cycle. The same pattern was observed upon using different volumes of lysis buffer (5, 10, 20 mL; Fig. 2b, Supp. Fig. 5). One gram of wet cell pellet yielded around 120 mg total protein as determined by the modified Bradford assay.

### Formation of heat-induced aggregates (HIAs)

*Tk*AMPpase is derived from a thermostable archaeon with an optimum growth temperature of 85 °C^34^. Therefore, heat treatment is a valuable tool to purify *Tk*AMPpase. To explore this strategy, we subjected crude preparations of the soluble protein (obtained after the fifth French press cycle) to 80 °C for 30 min. Thermal treatment of the soluble crude extract containing *Tk*AMPpase led to almost complete precipitation of *Tk*AMPpase (Fig. 2c). To study this effect in more detail, the impact of temperature on the enzyme’s solubility and the formation of aggregates was evaluated. Therefore, the soluble crude extract containing *Tk*AMPpase was incubated at six temperatures (from 40 to 90 °C in 10 °C increments) and aggregate formation was assessed every 15 min for 1 h. Room temperature (approximately 25 °C) was used to evaluate spontaneous aggregation. The relative amount of the formed aggregates was calculated as a percentage of the total *Tk*AMPpase amount (soluble and insoluble) of the protein (Fig. 2d, Supp. Fig. 6). At the lower tested temperatures (40 °C, 50 °C and 60 °C), an increase of the percentage of aggregates over time was observed. While a steady increase corresponding to an aggregation rate of 0.84 h^−1^ was monitored at 40 °C (Supp. Fig. 7), the aggregation at higher temperatures quickly reached a plateau of around 75% (50 °C) and 80% (60 °C). At even higher temperatures (70–90 °C), 80% aggregation was already detectable after 15 min with no significant change within the remaining hour (Fig. 2d). The aggregation observed at 25 °C was negligible (< 5%) and did not increase over time. We attribute these observations to the formation of thermally stable multimers of *Tk*AMPpase which aggregate as insoluble proteins. Our results reveal that this aggregation happens at temperatures as low as 40 °C with a highly temperature-dependent rate.

### Thermal stability analysis of the purified *Tk*AMPpase

**Figure 3:**
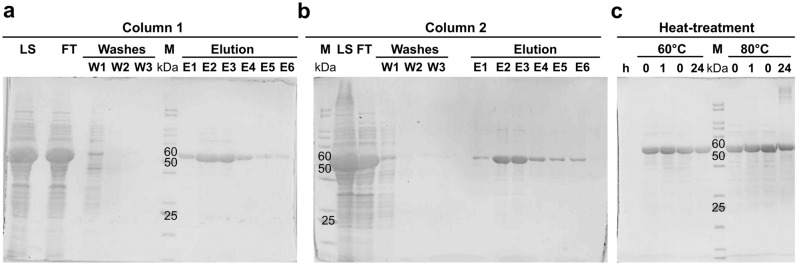
Ni-NTA purification of His-tagged *TK*AMPpase and its thermal stability. (**a**,**b**) 12% SDS-polyacrylamide gels showing the samples taken during the purification of *TK*AMPpase. Loaded sample (LS), flowthrough (FT), wash fractions (W1, W2, W3) and elution fractions (E1–E6). The FT from column 1 was loaded consecutively to column 2. (**c**) Heat treatment of the purified *Tk*AMPpase by incubation at 60 °C and 80 °C for 1 h and 24 h. A sample was taken before incubation (0 h).

In previous reports, a high thermal stability of purified *Tk*AMPpase was described^23^. Therefore, we evaluated the thermal stability of the Ni-NTA purified protein. Thus, using the described cell disruption methods, the N-terminal His-tagged *Tk*AMPpase was purified using a Ni-NTA agarose resin. As only few amounts of the protein bound to the column despite the high column capacity (250 mg of protein) another column was loaded with the flow-through of the first purification step. Nonetheless, *Tk*AMPpase remained mostly in the flowthrough (Fig. 3a,b). 1 g of cell pellet yielded around 6.9 mg of purified *Tk*AMPpase as determined by modified Bradford assay.

The purified *Tk*AMPpase was stable at 60 °C and at 80 °C for 1 h as determined by SDS-PAGE (Fig. 3c) and densitometric analysis. After 24 h, however, about 10% and 30% of the protein was precipitated at 60 °C and 80 °C, respectively, as determined by the densitometric analysis of the SDS-polyacrylamide gels which is much less than the aggregation observed from the crude extract (80% at 60 °C and 80 °C after 1 h, Fig. 2d). The higher rate of *Tk*AMPpase precipitation from the crude extract could be attributed to the crowded environment of the crude extract together with the presence/abundance of partially unfolded proteins (from *E. coli* or from the target protein) as a result of heat treatment. Such an environment facilitates the initiation and progression of protein assembly^35,36^. Furthermore, protein concentrations are much less in purified protein preparations compared to crude extracts, which might also influence protein aggregation.

### Specific activity of the HIA using CMP as a substrate

As it has been reported that *Tk*AMPpase multimers show activity towards a variety of 5′-mononucleotides^23^, we questioned if the formed HIAs retained phosphorolytic activity. Based on previously reported experimental data^22^, we used the phosphorolysis of CMP as a model reaction to determine the specific activity of the HIAs at 80 °C. These experiments revealed that all HIAs have catalytic activity with values of 0.08–0.2 U mg^−1^ (Fig. 2e). The HIAs obtained through aggregation between 40 and 60 °C showed no significant differences in their specific activities. In contrast, the aggregates formed at temperatures between 70 and 90 °C displayed a gradual decrease in their specific activity with the lowest activity observed after aggregation at 90 °C (Fig. 2e, Supp. Fig. 8). These data suggest that with increasing temperature more *Tk*AMPpase is denatured. However, full denaturation of *Tk*AMPpase was not observed under the applied conditions and all HIA preparations retained activity.

### Purification of CatIBs

**Figure 4:**
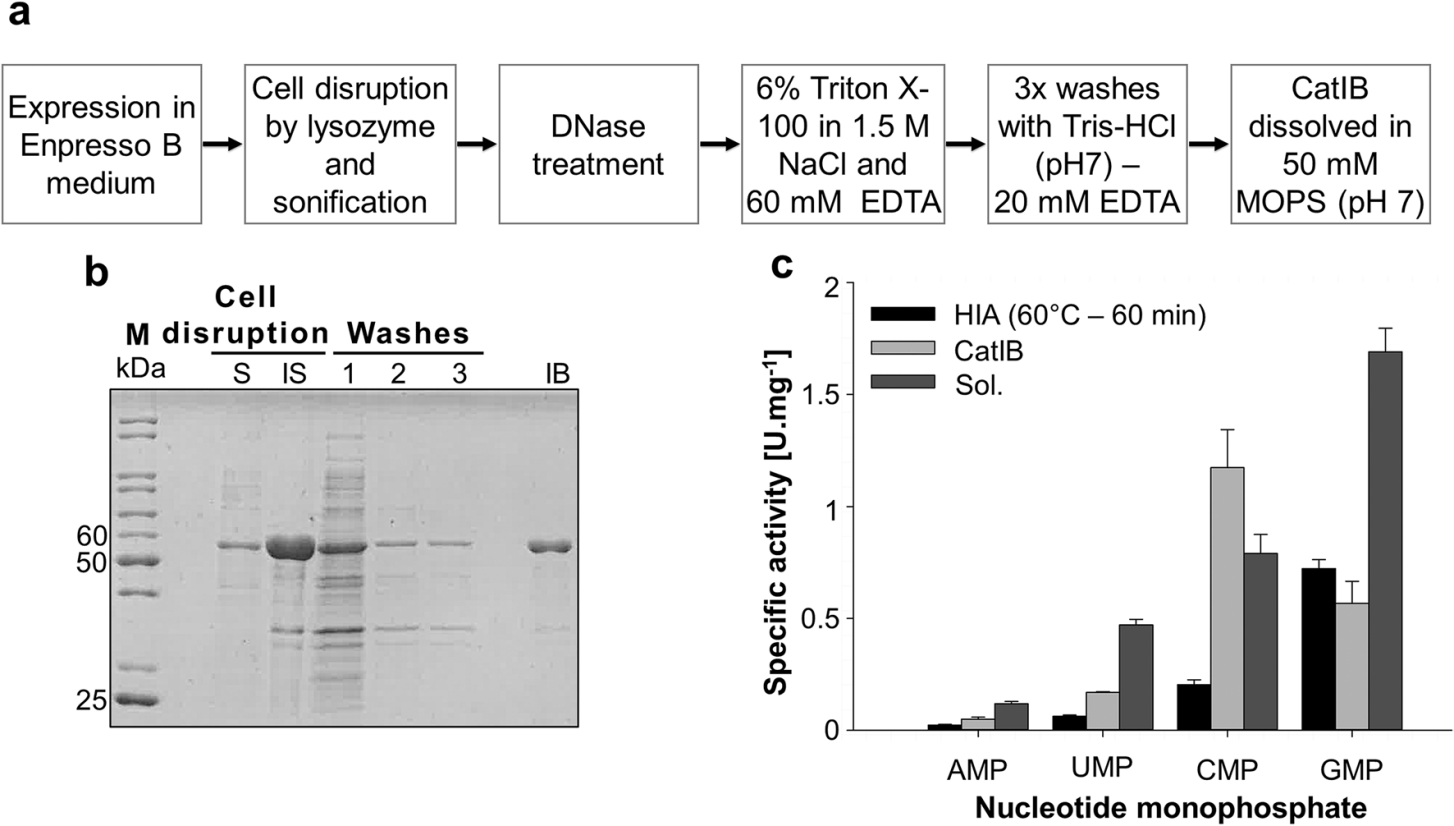
Expression and purification of CatIBs. (**a**) The utilized protocol for IB isolation. (**b**) 12% SDS-polyacrylamide gels showing the different steps of IB isolation. After cell disruption using sonification *Tk*AMPpase was expressed mainly in the insoluble fraction (IS) (2 µL of 1:10 dilution was used on the gel). Washing steps with Tris–HCl buffer were applied to decrease protein background (10 µL from each (W1, W2 and W3) was loaded on the SDS PAGE). The final IB preparation showed only minor impurities (IB) (2 µL of IB in a 1:16 dilution was loaded on the SDS-PAGE). (**c**) Specific activity of the CatIBs compared to the HIAs obtained from 60 °C heat treatment after 60 min was determined. The used reaction conditions were 2 mM substrate (NMP) and 50 mM potassium phosphate in MOPS buffer (pH 7) at 80 °C. Regular samples were taken at different time points over a period of 15 min. Standard deviation is derived from three experiments. *M* protein ladder, *IS* insoluble protein fraction, *S* soluble protein fraction. Cell disruption method: sonification.

Since *Tk*AMPpase is mainly expressed in the insoluble fraction and even aggregated protein showed activity in a model reaction, IB isolation seemed like an attractive alternative for protein purification. Therefore, we attempted to isolate active IBs of *Tk*AMPpase after expression in *E. coli*. To avoid the disruption of the *in-vivo* formed IB, sonication was used as a gentler cell disruption method compared to the French Press treatment applied before. This method yielded intact in-vivo formed IBs which were successfully purified directly from the insoluble protein fraction (Fig. 4a,b). 1 g of wet cell pellet yielded approximately 0.5 g of IBs (wet weight) which contained approximately 38 mg *Tk*AMPpase per gram cell pellet (wet weight; Supp. Fig. 4), which is about 10 times the amounts obtained from the Ni-NTA purification.

### Specific activity of CatIBs of *Tk*AMPpase in comparison to HIAs and purified protein

Initially, the activity of the *Tk*AMPpase IB was studied using CMP as substrate. A substrate conversion of 26% was recorded. To prove that the observed activity is not based on dissolved *Tk*AMPpase from the IB, preparations were incubated at 60 °C or 80 °C for 2.5 h. IB were then collected by centrifugation. To fully remove traces of IBs, the soluble fraction was additionally filtered through a 0.45 µm filter. AMPpase activity of the soluble fraction and the collected IB was analyzed at 80 °C. Almost no activity was detected for the supernatant of the 80 °C incubated preparation (0.1%), whereas a negligible activity (1.9%) was observed for the ones incubated at 60 °C. In contrast, the collected IB from 80 °C and 60 °C showed substrate conversions of 13% and 18%, respectively (Supp. Fig. 9).

Next, we compared the activity of the purified IBs of *Tk*AMPpase to the activity of the His-tag purified enzyme and HIAs formed at 60 °C after 1 h (Fig. 4c). AMP, GMP, CMP and UMP were applied as substrates in the presence of phosphate at 80 °C. The heat induced aggregates showed the lowest activity among the three different *Tk*AMPpase preparations. The isolated IBs showed two- to sixfold higher specific activities towards all substrates compared to the HIAs except for GMP (Fig. 4c). However, our CatIBs displayed lower specific activities (2.5–3 folds) compared to the His-tag purified *Tk*AMPpase for all tested substrates except for CMP. CatIBs specific activity for CMP was 1.5 and 6 folds higher than those of the His-tag purified enzyme and the HIA preparation, respectively. The specific activities of the three preparations were lower than those previously reported^22^, however reaction set up was different. We used a high ratio of phosphate to substrate (25:1), whereas previous reports used an equimolar phosphate and substrate concentration.

Interestingly, a comparison between the activities of different enzyme preparations (HIA, CatIB and soluble enzyme) revealed a difference in the substrate preferences. The CatIBs showed the highest activity with CMP, while GMP is the preferred substrate for HIAs and the soluble *Tk*AMPpase (Supp. Fig. 10). Altered substrate preferences is additionally observed as compared to published activity of the soluble enzyme where AMP was the second most preferred substate^22^, while, our three different preparation have the least activity towards AMP. There are increasing evidences that aggregates formed under different conditions (including different temperatures) display morphological differences^37–39^, with differences in secondary and tertiary structure additionally altering their function^38^. Since rather little is known about AMPpases, this class of enzymes requires further studies to develop a better understanding of their structure function relationships and aggregation behavior.

## Conclusion

*Tk*AMPpase belongs to the small group of enzymes that naturally forms CatIBs without an artificial tag during its heterologous expression in *E. coli*. Whereas high pressures enabled obtaining the enzyme in the soluble fraction, heat treatment of the crude soluble extract at various temperatures induces the re-formation of insoluble aggregates. The CatIBs were highly active and were obtained in high quantities as compared to the in-vitro heat induced aggregates and to the His-tag purified enzyme. Although further work is necessary to gain a better understanding of the substrate spectrum of *Tk*AMPpase as well as the reasons for the observed aggregation, the results presented in this study encourage exploration of this enzyme as a self-immobilizing biocatalyst for applications in heterogenous reaction systems.

## Supplementary Information

Supplementary Information.

## Abbreviations

AMP: Adenosine 5′-monophosphate
*Tk*AMPpase: *Thermococcus kodakarensis* Adenosine 5′-monophosphate phosphorylase
CatIB: Catalytically active inclusion body
CMP: Cytidine 5′-monophosphate
GMP: Guanosine 5′-monophosphate
HIA: Heat-induced aggregate
IB: Inclusion body
NMP: Nucleoside 5′-monophosphate
dNMP: 2′-Deoxy-nucleoside 5′-monophosphate
R15P: Ribose-1,5-bisphosphate
UMP: Uridine 5′-monophosphate

## References

[1] Krauss U, Jäger VD, Diener M, Pohl M, Jaeger KE (2017). Catalytically-active inclusion bodies—Carrier-free protein immobilizates for application in biotechnology and biomedicine. J. Biotechnol..

[2] Rinas U (2017). Bacterial inclusion bodies: Discovering their better half. Trends Biochem. Sci..

[3] Ventura S (2005). Sequence determinants of protein aggregation: Tools to increase protein solubility. Microb. Cell Fact..

[4] Rodríguez-Bolaños M, Miranda-Astudillo H, Pérez-Castañeda E, González-Halphen D, Perez-Montfort R (2020). Native aggregation is a common feature among triosephosphate isomerases of different species. Sci. Rep..

[5] Ventura S, Villaverde A (2006). Protein quality in bacterial inclusion bodies. Trends Biotechnol..

[6] Villaverde A, Carrió MM (2003). protein aggregation in recombinant bacteria: Biological role of inclusion bodies. Biotechnol. Lett..

[7] Wang L (2009). Towards revealing the structure of bacterial inclusion bodies. Prion.

[8] Singh A, Upadhyay V, Singh A, Panda AK (2020). Structure–function relationship of inclusion bodies of a multimeric protein. Front. Microbiol..

[9] Garcı E (2012). Bacterial inclusion bodies: Making gold from waste. Trends Biotechnol..

[10] Neubauer P, Fahnert B, Lilie H, Villaverde A, Shively JM (2006). Protein inclusion bodies in recombinant bacteria. Inclusions in Prokaryotes.

[11] Jäger VD (2020). Catalytically-active inclusion bodies for biotechnology—General concepts, optimization, and application. Appl. Microbiol. Biotechnol..

[12] Jäger VD (2019). Tailoring the properties of (catalytically)-active inclusion bodies. Microb. Cell Fact..

[13] Kloss R (2018). Catalytically active inclusion bodies of l-lysine decarboxylase from *E. coli* for 1,5-diaminopentane production. Sci. Rep..

[14] Nahálka J, Pätoprstý V (2009). Enzymatic synthesis of sialylation substrates powered by a novel polyphosphate kinase (PPK3). Org. Biomol. Chem..

[15] Diener M, Kopka B, Pohl M, Jaeger KE, Krauss U (2016). Fusion of a coiled-coil domain facilitates the high-level production of catalytically active enzyme inclusion bodies. ChemCatChem.

[16] García-Fruitós E (2005). Aggregation as bacterial inclusion bodies does not imply inactivation of enzymes and fluorescent proteins. Microb. Cell Fact..

[17] Mestrom L (2019). Artificial fusion of mCherry enhances trehalose transferase solubility and stability. Appl. Environ. Microbiol..

[18] Dong Q, Yan X, Zheng M, Yang Z (2014). Characterization of an extremely thermostable but cold-adaptive β-galactosidase from the hyperthermophilic archaeon *Pyrococcus**furiosus* for use as a recombinant aggregation for batch lactose degradation at high temperature. J. Biosci. Bioeng..

[19] Tokatlidis K, Dhurjati P, Millet J, Béguin P, Aubert J-P (1991). High activity of inclusion bodies formed in *Escherichia**coli* overproducing *Clostridium**thermocellum* endoglucanase. FEBS J..

[20] Worrall DM, Goss NH (1989). The formation of biologically active beta-galactosidase inclusion bodies in *Escherichia**coli*. Aust. J. Biotechnol..

[21] Sato T, Atomi H, Imanaka T (2007). Archaeal type III RuBisCOs function in a pathway for AMP metabolism. Science.

[22] Aono R (2012). Enzymatic characterization of amp phosphorylase and ribose-1,5-bisphosphate isomerase functioning in an archaeal amp metabolic pathway. J. Bacteriol..

[23] Nishitani Y (2013). Structure analysis of archaeal AMP phosphorylase reveals two unique modes of dimerization. J. Mol. Biol..

[24] Sato T, Atomi H, Imanaka T (2007). Supporting data_Archaeal type III RuBisCOs function in a pathway for AMP metabolism. Science.

[25] Szeker K, Niemitalo O, Casteleijn MG, Juffer AH, Neubauer P (2010). High-temperature cultivation and 5′ mRNA optimization are key factors for the efficient overexpression of thermostable *Deinococcus**geothermalis* purine nucleoside phosphorylase in *Escherichia coli*. J. Biotechnol..

[26] Šiurkus J (2010). Novel approach of high cell density recombinant bioprocess development: Optimisation and scale-up from microlitre to pilot scales while maintaining the fed-batch cultivation mode of *E. coli* cultures. Microb. Cell Fact..

[27] Sambrook J, Russell DW (2001). Molecular Cloning: A Laboratory manUal.

[28] Schneider CA, Rasband WS, Eliceiri KW (2012). NIH Image to ImageJ: 25 years of image analysis. Nat. Methods.

[29] Kaspar F (2019). A UV/Vis spectroscopy-based assay for monitoring of transformations between nucleosides and nucleobases. Methods Protoc..

[30] Kaspar F (2020). Spectral unmixing-based reaction monitoring of transformations between nucleosides and nucleobases. ChemBioChem.

[31] Singh A, Upadhyay V, Upadhyay AK, Singh SM, Panda AK (2015). Protein recovery from inclusion bodies of *Escherichia**coli* using mild solubilization process. Microb. Cell Fact..

[32] Boonyaratanakornkit BB, Park CB, Clark DS (2002). Pressure effects on intra- and intermolecular interactions within proteins. Biochim. Biophys. Acta Protein Struct. Mol. Enzymol..

[33] Gross M, Jaenicke R (1994). Proteins under pressure: The influence of high hydrostatic pressure on structure, function and assembly of proteins and protein complexes. Eur. J. Biochem..

[34] Atomi H, Fukui T, Kanai T, Morikawa M, Imanaka T (2004). Description of *Thermococcus**kodakaraensis* sp. nov., a well studied hyperthermophilic archaeon previously reported as *Pyrococcus* sp. KOD1. Archaea.

[35] Schramm FD, Schroeder K, Jonas K (2019). Protein aggregation in bacteria. FEMS Microbiol. Rev..

[36] Wang W, Nema S, Teagarden D (2010). Protein aggregation—pathways and influencing factors. Int. J. Pharm..

[37] Jung JM, Savin G, Pouzot M, Schmitt C, Mezzenga R (2008). Structure of heat-induced β-lactoglobulin aggregates and their complexes with sodium-dodecyl sulfate. Biomacromol.

[38] Natalello A, Santarella R, Doglia SM, de Marco A (2008). Physical and chemical perturbations induce the formation of protein aggregates with different structural features. Protein Expr. Purif..

[39] Shivu B (2013). Distinct β-sheet structure in protein aggregates determined by ATR-FTIR spectroscopy. Biochemistry.

