# Supplementary material for: Thermostable adenosine 5′-monophosphate phosphorylase from Thermococcus kodakarensis forms catalytically active inclusion bodies
Source: Sci Rep. 2021 Aug 19;11:16880. doi: 10.1038/s41598-021-96073-5 (PMC8376864; doi:10.1038/s41598-021-96073-5)
Supplement: Supplementary file 1 — Supplementary Information. [file 41598_2021_96073_MOESM1_ESM.pdf]

Supplementary Material

Thermostable Adenosine 5'-Monophosphate Phosphorylase from *Thermococcus kodakarensis* forms catalytically active inclusion bodies

Sarah Kamel[1‡], Miriam C. Walczak[1,2‡], Felix Kaspar[1,2], Sarah Westarp[1,2], Peter Neubauer[1] and Anke Kurreck[1,2*]

[1] Technische Universität Berlin, Chair of Bioprocess Engineering, Straße des 17. Juni 135, 10623 Berlin, Germany

[2] BioNukleo GmbH, Ackerstraße 76, 13355 Berlin, Germany

‡The authors contributed equally to the work

*Corresponding author

Supp. table 1. Cell lysis methods employed in this work.

Lysis method	Purpose of use	Type of lysis	Composition of the buffer	Amount of the buffer used	Weight of the cell pellet used
BugBuster	Checking the expression	Enzymatic	- BugBuster reagent<br>- lysozyme (final conc. of 50 µg/mL)<br>- DNAase (final conc. of 1 µg/mL)<br>- $MgCl_2$ (final conc. of 1 mM)	12 mL/ g cell pellet	25 mg
French press	Obtaining soluble protein as crude extract	Mechanical	- **Buffer 1:**<br>- 0.1 M Tris-HCl buffer (pH 7)<br>- 1 mM EDTA<br>- **Buffer 2 (after cell disruption):**<br>- 1.5 M NaCl (pH 7)<br>- 60 mM EDTA,<br>- 6% Triton-X100<br>- 0.1 mM PMSF	5 mL/g cell pellet	4 g for each experiment
French press	His-tag purification	Enzymatic and mechanical	- **Lysis buffer:**<br>- 50 mM sodium phosphate (pH 7)<br>- 300 mM NaCl<br>- 10 mM Imidazole<br>- lysozyme (final conc. of 50 µg/mL)<br>- DNAase (final conc. of 1 µg/mL)<br>- $MgCl_2$ (final conc. of 1 mM)	5 mL/g cell pellet	2 g
Sonication	Purification of the inclusion bodies	Enzymatic and mechanical	- **Buffer 1:**<br>- 0.1 M Tris-HCl buffer (pH 7)<br>- 1 mM EDTA<br>- lysozyme (final conc. of 1.5 mg/mL)<br>After mechanical cell disruption:<br>- DNase (final concentration of 50 µg)<br>- 3 mM $MgCl_2$<br>- 0.1 mM PMSF<br>- **Buffer 2 (after cell disruption):**<br>- 1.5 M NaCl (pH 7)<br>- 60 mM EDTA,<br>- 6% Triton-X100	5 mL/g cell pellet	2 g for each experiment

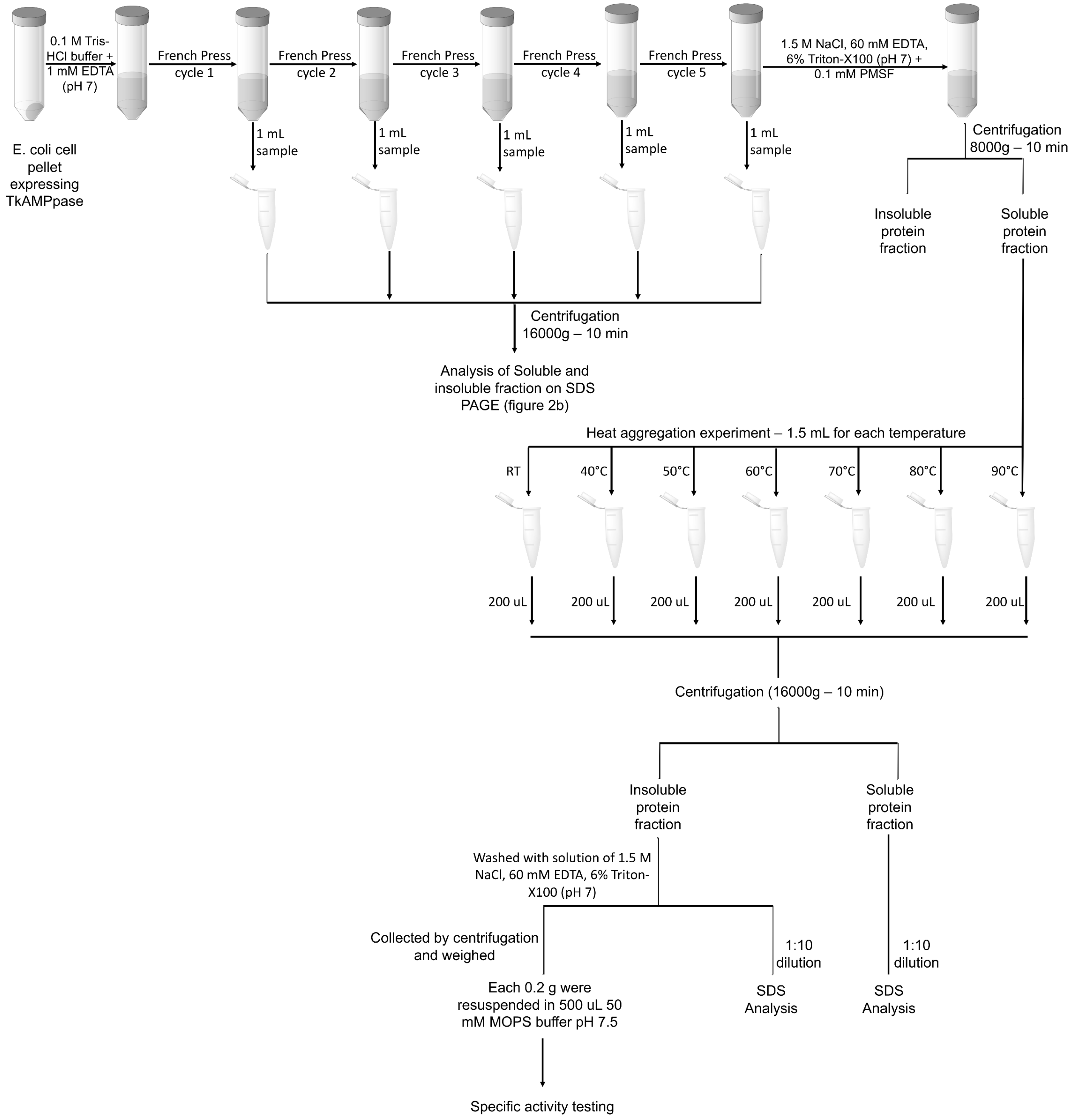

Supp. fig. 1. Schematic presentation of the Isolation of *Tk*AMPpase as soluble crude protein extract using French press and the consequent heat induced aggregation.

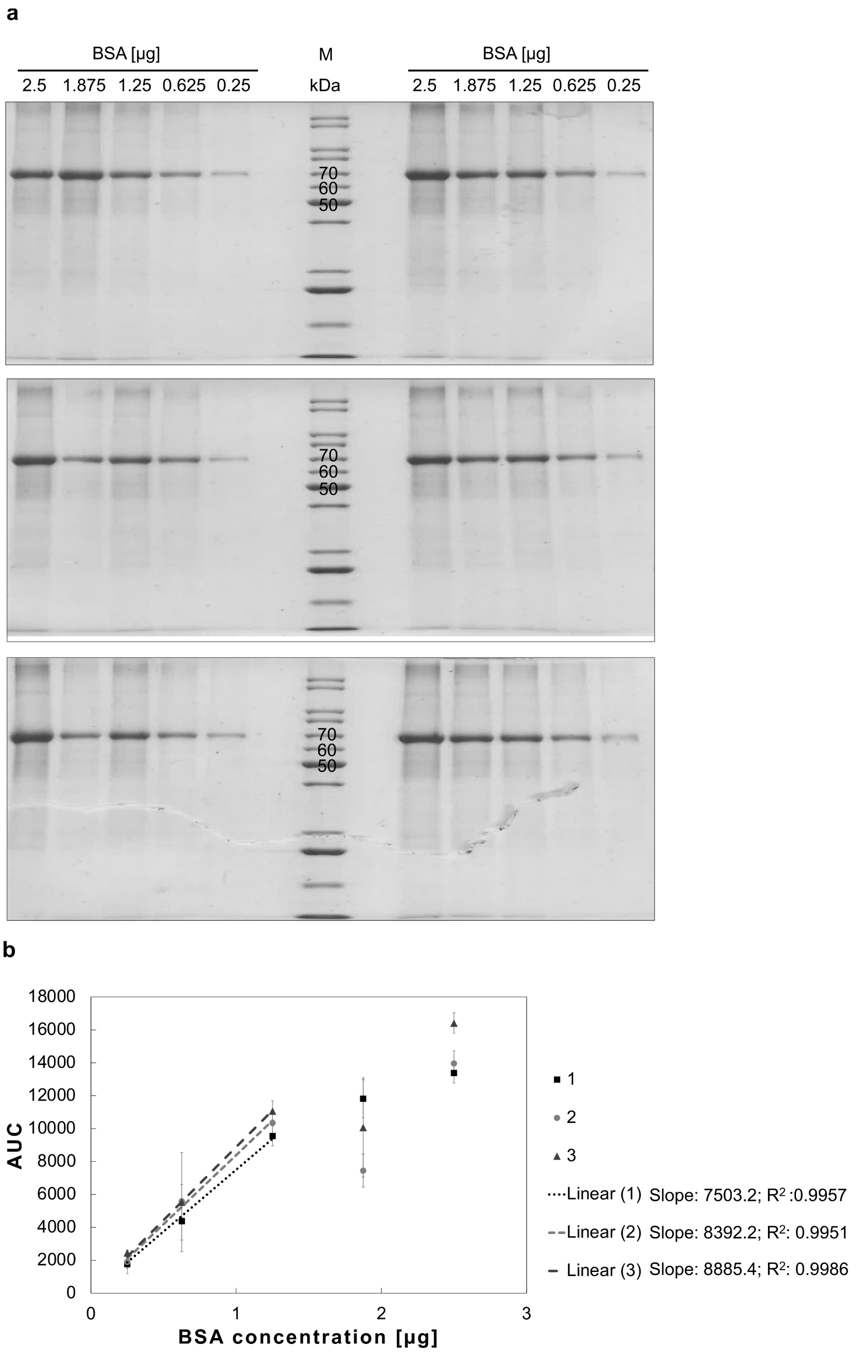

Supp. fig. 2. Calibration curve for the BSA reference concentrations **(a)** For protein quantification 12% SDS-PAGE was used. Five different BSA-concentrations are used as duplicates (2.5 – 0.25 µg) and are detected at 70 kDa, approximately. **(b)** The intensity of the bands was measured using ImageJ software and the area under the curve (AUC) was determined and plotted. The intensity of the two highest amounts of BSA (2.5, 1.875 µg) were not in the linear range. Thus, for further protein quantification BSA-standards of 1.25 µg, 0.625 µg and 0.25 µg were used.

a

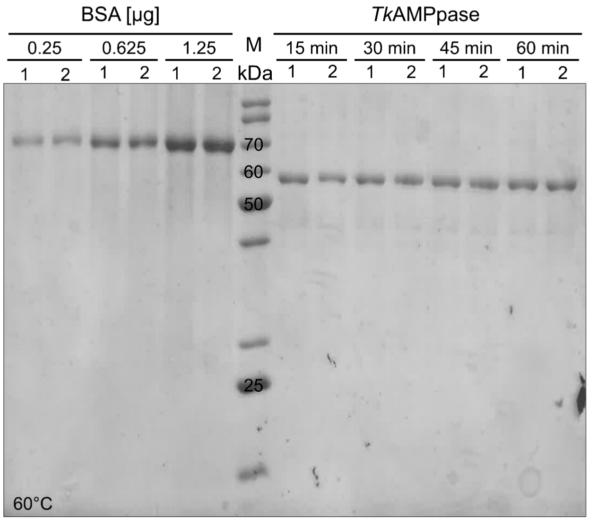

b

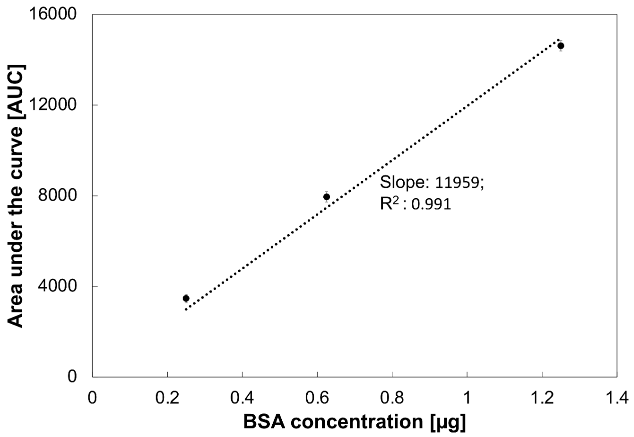

c

Temp. [°C]	Duration [min]	AUC for the HIA				Protein conc. [mg/mL]			
		1	2	Average	SD	1	2	Average	SD
60	15	12037.6	11838.1	11937.9	99.7	2.52	2.47	2.50	0.02
	30	10954.2	10456.2	10705.2	249	2.29	2.19	2.24	0.05
	45	9303.3	8940.9	9122.1	181.1	1.94	1.87	1.91	0.04
	60	6890.7	9483.1	8186.9	1296.2	1.44	1.98	1.71	0.27

Supp. fig. 3. Protein quantification of different *Tk*AMPpase HIAs at 60°C as an example. **(a)** For protein quantification 12% SDS-PAGE was used. Three different BSA-preparations as duplicates are visible on the gels (1.25 – 0.25 µg) at 70 kDa and four different preparations as duplicates of HIAs at 60°C for 15, 30, 45 and 60 min at 56 kDa, approximately. **(b)** The intensity of the BSA-bands were analyzed using ImageJ to calculate the slope. Area under the curves (AUC) of the three BSA concentrations were used for the generation of the calibration curve. **(c)** The intensity of the four different preparations of HIAs bands were analyzed and the AUC was determined. The protein concentration was calculated by dividing the measured AUC value by the slope; taking into consideration the dilution of the protein samples with loading buffer and the volume loaded on the gel.

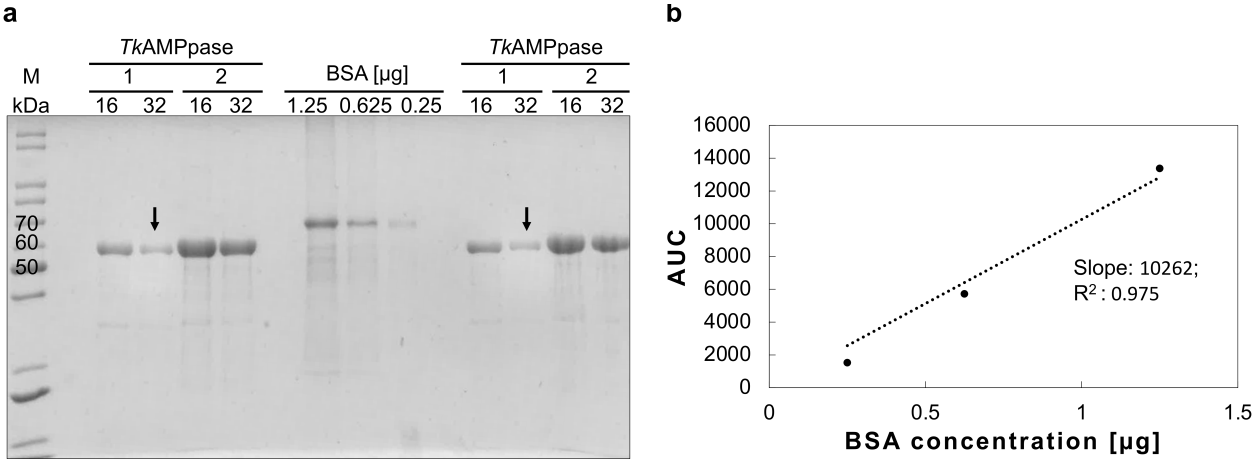

c

CatIB [replicas]	AUC			Protein conc. [mg/mL]		
		Average	SD		Average	SD
1	5280.7	4805.1	672.7	1.3	1.2	0.1
2	4329.4			1.1		

Supp. fig. 4. Protein quantification of different *Tk*AMPpase CatIB using 12%SDS-PAGE **(a)** Three different BSA-concentrations were loaded and detected on the gels (1.25 – 0.25 µg) at 70 kDa, approximately. Two different IB preparations (1 and 2) were loaded in duplicate and identified on the gel at 54 kDa, approximately. The purified IB-preparation were diluted 1:16 and 1:32 to identify the better quantifiable amount within the linear range of the standard BSA. Only the first preparation (as shown by arrows) was used for the quantification and for later activity testing. **(b)** The intensity of the BSA-bands was analyzed using ImageJ to calculate the slope and the area under the curves (AUC) of the three BSA concentrations were plotted to generate the calibration curve. **(c)** The intensity of the bands was analyzed, and the AUC was determined. The protein concentration was calculated by dividing the measured AUC value by the slope; taking into consideration the dilution of the protein samples with loading buffer and the volume loaded on the gel.

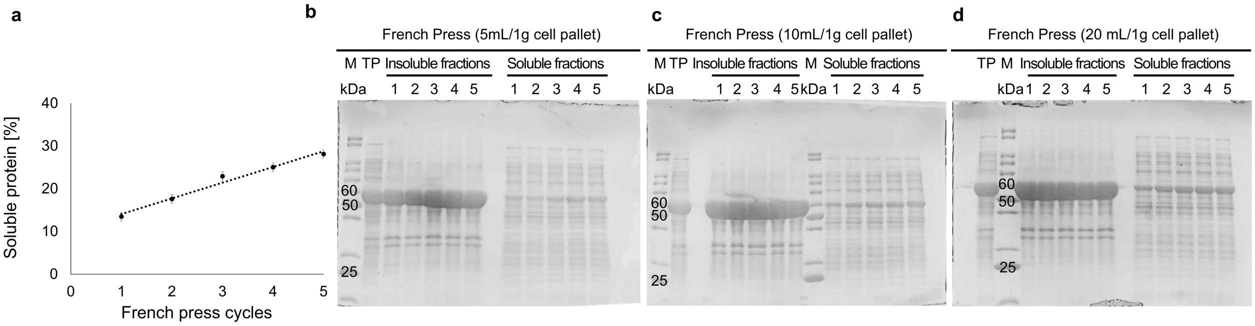

Supp. fig. 5. Purification of *Tk*AMPpase using French Press. (**a**)The quantification of the bands on the 12% SDS PAGE was done using ImageJ software. (**b, c, d**) French press cell disruption using 5-, 10- and 20-mL lysis buffer respectively. SDS-PAGE showing the soluble (S) and insoluble (IS) fraction after each French Press cycle for 5 consecutive cycles.

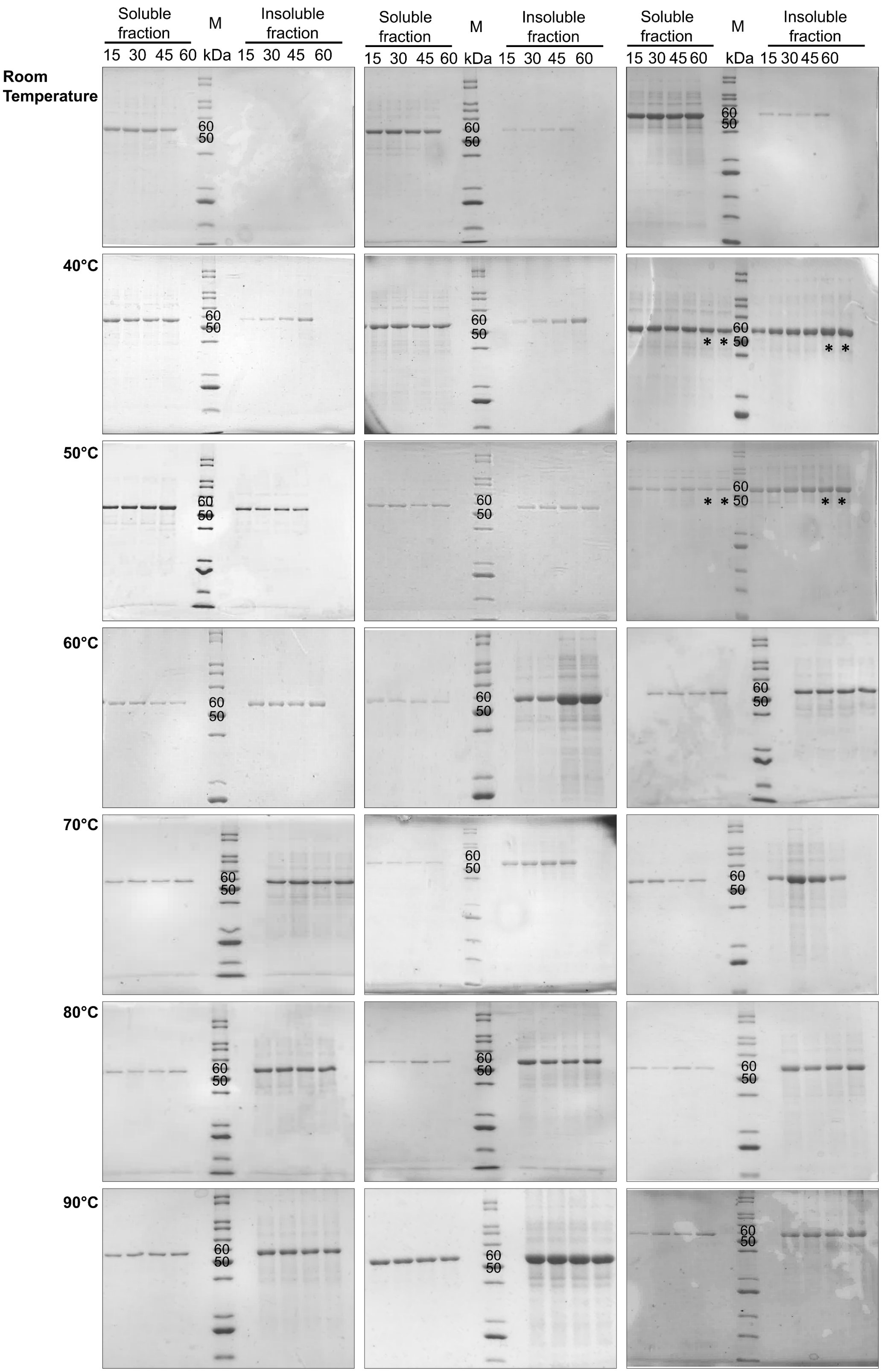

Supp. fig. 6. SDS-PAGE of the HIAs formed at all the experimented temperature and their counterpart soluble fraction. At 40°C and 50°C extra samples were (*) taken after 90 min and 120 min to assess if further incubation would increase the aggregation.

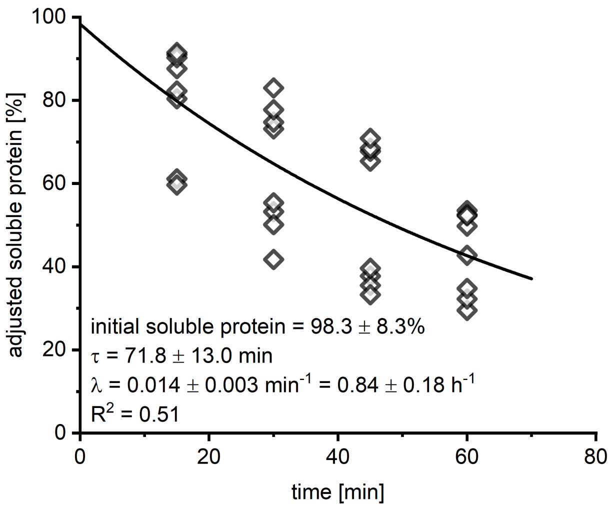

Supp. fig. 7. Aggregation rate of *Tk*AMPpase at 40 °C. The data shown in Figure 1d of the main text were used to approximate the aggregation rate by normalization to a maximum aggregation percentage of 80% and fitting of the resulting data for the soluble protein as a first order exponential decay with variable initial values and mean aggregation time τ. The aggregation rate constant λ was obtained as the inverse of τ.

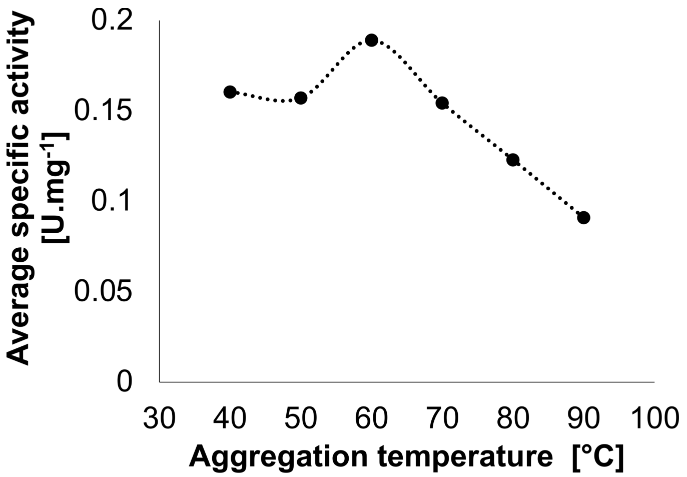

Supp. fig. 8. Average specific activity of the four time points in every tested temperature (figure 1e).

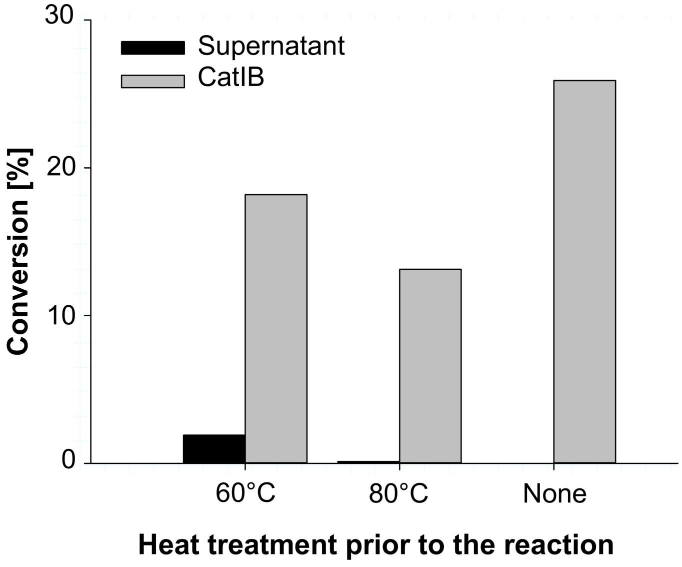

Supp. fig. 9. 1 mL of 1 mg.mL^{-1} CatIB was incubated at 60 and 80°C for 2.5h followed by centrifugation. The supernatant was filtered through 0.45 µm syringe filter, while the collected CatIB were re-suspended in 1 mL 50 mM MOPS buffer. The heat treated supernatant and CatIB and un-heat treated CatIB were used to test for their catalytic activity. CMP was used as a substrate and the reaction was performed under the same standard conditions (2mM substrate – 50 mM MOPS – 50 mM phosphate – at 80°C for 3 mins). Conversion % is defined as the percentage of substrate converted to product.

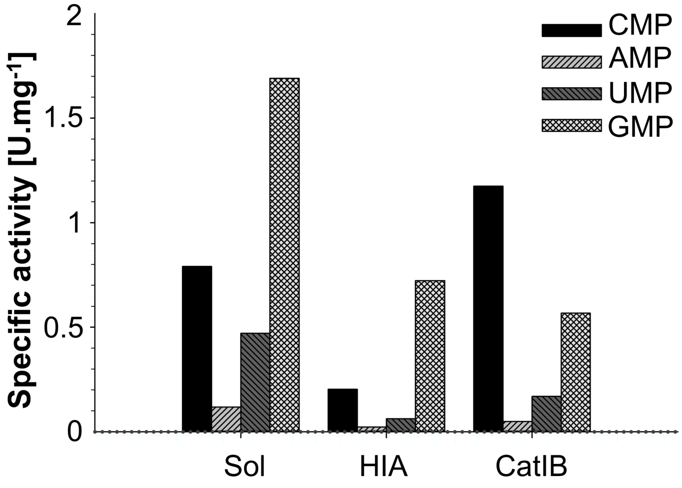

Supp. fig. 10. Substrate preference of the different enzyme preparations; heat induced aggregates (HIA) and catalytically active inclusion bodies (CatIBs) reactions were performed as mentioned above.

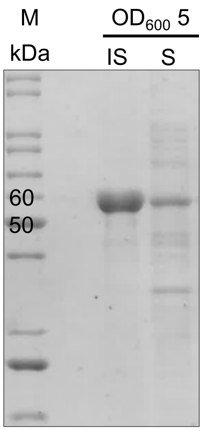

Supp. fig. 11. The complete SDS PAGE of OD_{600} =5 shown in Fig. 2 of the manuscript
